# Residual efficiency of iron-nanoparticles and different iron sources on growth, and antioxidants in maize plants under salts stress: life cycle study

**DOI:** 10.1016/j.heliyon.2024.e28973

**Published:** 2024-04-01

**Authors:** Hameed Alsamadany, Sidra Anayatullah, Muhammad Zia-ur-Rehman, Muhammad Usman, Talha Ameen, Hesham F. Alharby, Basmah M. Alharbi, Awatif M. Abdulmajeed, Jean Wan Hong Yong, Muhammad Rizwan

**Affiliations:** a Department of Biological Sciences, Faculty of Science, King Abdulaziz University, Jeddah, 21589, Saudi Arabia; b Plant Biology Research Group, Department of Biological Sciences, Faculty of Science, King Abdulaziz University, Jeddah, 21589, Saudi Arabia; c Institute of Soil and Environmental Sciences, University of Agriculture Faisalabad, Pakistan; d Biology Department, Faculty of Science, University of Tabuk, Tabuk, 71491, Saudi Arabia; e Biology Department, Faculty of Science, University of Tabuk, Umluj, 46429, Saudi Arabia; f Department of Biosystems and Technology, Swedish University of Agricultural Sciences, 23456, Alnarp, Sweden; g Department of Environmental Sciences, Government College University Faisalabad, Faisalabad, 38000, Pakistan

**Keywords:** Salt stress, Maize, Iron nanoparticles, Peroxidase, Carotenoids, Nutrients, Residual, Photosynthesis

## Abstract

Exogenous application of iron (Fe) may alleviate salinity stress in plants growing in saline soils. This comparative study evaluated the comparative residual effects of iron nanoparticles (FNp) with two other Fe sources including iron-sulphate (FS) and iron-chelate (FC) on maize (*Zea mays* L.) crop grown under salt stress. All three Fe sources were applied at the rate of 15 and 25 mg/kg of soil before the sowing of wheat (an earlier crop; following the sequence of crop rotation) and no further Fe amendments were added later for the maize crop. Results revealed that FNp application at 25 mg/kg (FNp-2) substantially increased maize height, root length, root dry weight, shoot dry weight, and grain weightby 80.7%, 111.1%, 45.7%, 59.5%, and 77.2% respectively, as compared to the normal controls; and 62.6%, 81.3%, 65.1%, 78%, and 61.2% as compared to salt-stressed controls, respectively. The FNp-2 treatment gave higher activities of antioxidant enzymes, such as superoxide dismutase, peroxidase, catalase, and ascorbate peroxidase compared to salt stressed control (50.6%, 51%, 48.5%, and 49.2%, respectively). The FNp-2 treatment also produced more photosynthetic pigments and better physiological markers: higher chlorophyll *a* contents by 49.9%, chlorophyll *b* contents by 67.2%, carotenoids by 62.5%, total chlorophyll contents by 50.3%, membrane stability index by 59.1%, leaf water relative contents by 60.3% as compared to salt stressed control. The highest Fe and Zn concentrations in maize roots, shoots, and grains were observed in FNp treatment as compared to salts stressed control. Higher application rates of Fe from all the sources also delivered better outcomes in alleviating salinity stress in maize compared to their respective low application rates. The study demonstrated that FNp application alleviated salinity stress, increased nutrient uptake and enhanced the yield of maize grown on saline soils.

## Highlights

• Residual effects of three Fe sources on salt tolerance and yield in maize crop were studied.
• Iron nanoparticles (FNp) delivered better responses in maize for growth, physiology, antioxidant enzyme activities, and nutrient uptake than iron sulphase (FS) and iron chelate (FC).
• The application of 25 mg/kg Fe sources to maize was more effective than 15 mg/kg under experimental conditions.

## 1 Introduction

Soil salinization is the main factor, which affects plant growth and development, covering 20% of irrigated lands worldwide [1,2]. In Pakistan, 64% yield losses have been reported due to salinization that covers ∼14% of irrigated lands leaving only ∼23 million hectares of area to meet sustainable agricultural activities [3]. The problem is becoming more severe due to anthropogenic activities, including irrigation with brackish water, desertification, over-grassing, excessive fertilization, and natural phenomena like low rainfall, high temperature, and weathering of soil minerals [4]. Moreover, the extremes in climate change like temperature abnormalities, rainfall intensity and patterns etc. may cause the genesis and expansion in salt-affected soils [[5], [6], [7]]. These factors alter the physiochemical properties of soils and significantly decrease the production potential, causing a serious threat to food security of the world [8].

Decreased soil quality, limits the efficiency of plants to uptake water and nutrients under the higher concentration of salt ions in the root zone, resulting in the reduced plant health at all stages such as seed germination, vegetative and reproductive [9]. Furthermore, crop productivity under salt stress is reduced through complex traits, including oxidative stress, osmotic stress, ionic toxicity, nutrients imbalance, hormonal imbalance, membrane dysfunction, and reduced photosynthetic activity [10]. Salt stress also damages the biological membrane integrity, cell wall, protein, DNA, and overall cell structure in plants through the excessive production of highly reactive oxygen species (ROS) such as superoxide (O^2−^) and hydrogen-peroxide [11]. Overproduction of ROS species may increase the sensitivity of plants towards salt stress by down regulating the activities of antioxidants with disturbed photosynthesis and increased cell death [12]. Iron (Fe) is an important element in crops because it is an essential component of many enzymatic processes, and proteins, involves in nucleic acid synthesis, and chlorophyll production [13,14]. Moreover, Fe has a crucial role in plant biochemical and physiological processes, including nitrogen fixation, photosynthesis, and respiration [[15], [71]]. The alteration in soil pH has been investigated in saline and alkaline soils, leading to the low solubility of Fe [16,17].

To address the dual problem of Fe deficiency and salinity stress, recent studies focused on the use of nanotechnology. The approach is environment-friendly and capable for improving crop productivity by increasing disease resistance, the uptake ability of nutrients, and reducing the application of pesticides and fertilizers [18]. Among the different nanoparticles, iron nanoparticles (FNp) might be the potential agent for plant development under salt-affected soils due to their small size, enhanced stability, biochemical reactivity, absorption, and unique physical properties [19]. Besides, compensating for plant Fe deficiency, reports indicated that FNp could reduce nutrient loss, enhance target delivery, and improve physiological processes, and antioxidant enzyme activities in plants due to their specific characteristics [20,21]. The ameliorative effects of FNp on maize [16], peppermint [22], grape [23], sorghum [24], strawberry [25], and wheat [18] grown under salt-affected soils, have been investigated. Although Fe sources have been successfully reported in the alleviation of salt stress in maize plants, little work is available pertaining to the residual effects of the Fe-based amendments on salt stress alleviation in the "next crops" along the lines of a typical wheat-to-maize cropping cycle [70].

Maize is known to be a moderately sensitive cereal crop to salinity (with a threshold salinity of 5 dS/m) which is a major environmental constraint hindering maize production with a grain loss of about 11% [8,26]. Germination rate and growth characteristics may be hindered by the hyperosmotic stress conditions induced by salinity leading to reduced overall yield [27]. In our previous study by Zia-ur-Rehman et al. [28], it was observed that FNp addition delivered better plant growth than conventional Fe sources including Fe chelate (Fe-EDTA) and Fe sulphate (FeSO_4_) under normal as well as saline soils. Moving forward, it is vital to understand the residual effects of these Fe sources and their long-term effects on yield and the key biological and soil parameters: photosynthesis, the antioxidant defense system, and the nutrient and water balance of soils affected by salinity. The current experiment was carefully planned to examine the residual effects of different Fe sources on various biological and soil parameters by the cultivation of maize after the harvesting of wheat crop.

## 2 Materials and methods

### 2.1 Study location and environmental conditions

The pot study was performed in the greenhouse of the University of Agriculture Faisalabad‌ (31°25′59.7″N 73°04′ 20.1″ E); the greenhouse is covered with glass panels, and provided with proper ventilation. The average temperature of the greenhouse was 38 ± 5 °C, average relative humidity was 65%, and a photoperiod of 14 h of daylight followed by 10 h of darkness, at the time of maize seed sowing. While the average temperature of the greenhouse was 28 ± 5 °C, and relative humidity was 75% at harvesting of maize crop.

### 2.2 Experimental material preparation and treatment application

The top layer (0–15 cm) of soil used for the study was collected from the research area at the Institute of Soil and Environmental Sciences (ISES). The initial physicochemical properties of the collected soil were analyzed as reported by Zia-ur-Rehman et al. [28].. The soil was spiked with a calculated amount of salts mixture to achieve the desired electrical conductivity (EC_e_) of 6 dS/m and sodium adsorption ratio (SAR) of 20 (mmol/L)^1/2^, Zia-ur-Rehman et al. [28] following the quadratic equation, method used by Mahmood et al. [29]. Briefly, the sieved soil was spiked with a mixture of salts: CaCl_2_ (166.5 mg/kg), NaCl (1404 mg/kg), MgCl_2_ (102.5 mg/kg), Na_2_SO_4_ (568.0 mg/kg), K_2_SO_4_ (34.8 mg/kg), Na_2_CO_3_ (53.0 mg/kg), and NaHCO_3_ (270.0 mg/kg), calculated using the quadratic equation. The soil was saturated with the measured salts dissolved in distilled water (half of the saturation percentage of soil; water was added) and incubated for 90 days at room temperature under field capacity. The soil analysis (soil saturated paste was prepared) showed pH of 7.5, carbonates of 0.75 meq/L, bicarbonates of 2.5 meq/L, chlorides of 7.95 meq/L, calcium plus magnesium was 8.55 meq/L, sodium was 7.75 meq/L, and potassium ion was 6.65 meq/L, before the spiking of salts in the soil. The soil was classified as sandy clay loam, comprising of 46% sand, 24% silt, and 30% clay contents, having saturation percentage of 35.5%. Ceramic pots with a diameter of 37 cm and a length of 42 cm were used for experimentation. Two levels (15 and 25 mg/kg) of three amendments; Fe nanoparticles (FNp), Fe-EDTA (FC), and FeSO_4_ (FS) were applied in the soil before the sowing of the earlier wheat crop (in a previous experiment [28], following the sequence of wheat-maize cropping [70]) by making solution in distilled water. The soil was added in the pots at the rate of 9 kg/pot and the pots were lined (on the inside) with plastic bags. The basic physicochemical properties of the applied amendments were reported by Zia-ur-Rehman et al. [28] where FNp has 97% purity, a surface area of 19–51 m^2^/g, a density of 5.1 g/m, and a particle-size range of 55–100 nm. Laboratory grade Fe-EDTA and FeSO_4_ were purchased from a scientific store at Faisalabad. A total of 7 treatments were designed: control {CNS (control of normal soil), CSS (control of saline-sodic soil)}, FNp-1 (15 mg/kg of FNp), FNp-2 (25 mg/kg of FNp), FC-1 (15 mg/kg of FC), FC-2 (25 mg/kg of FC), FS-1 (15 mg/kg of FS), and FS-2 (25 mg/kg of FS). Two types of soil; normal soil and salts spiked soil was used in the experiment. The design of experiment was completely randomized design (CRD). Each treatment involved three replications; thus, the experiment comprised of 42 pots.

### 2.3 Seed sowing and agronomic practices

Maize (*Zea mays* L.,variety Monsanto 6317) seeds were sown in randomly placed pots under controlled conditions and with soils taken after harvesting of the previous crop (wheat). Primarily, seven maize seeds were grown in each pot, subsequently thinned after 12 days of sowing, and maintained four plants per pot. To avoid nutrient deficiency, the soil was applied with a basal dose of nitrogen, phosphorous, and potassium at recommended rates of 160: 90: 60 kg/ha respectively. Soil application of diammonium phosphate (DAP as N, P source; 1.57 g/pot), and sulphate of potash (SOP as K source; 0.54 g/pot) was done completely before sowing, while urea (N source; 0.82 g/pot) was applied in two splits (1st dose at sowing; 0.41 g/pot, 2nd after 15 days of sowing that was vegetative stage; 0.41 g/pot). Distilled water was applied during the whole growth stage at a depth of 1–2 cm and grew for 120 days from July to October 2021.

### 2.4 Plant physiological and biochemical parameters

A portable and open gas exchange photosynthetic system was utilized to measure the foliar photosynthetic rate, stomatal conductance, gas exchange parameters, and transpiration rates [66]; the gas exchange calculations were in accordance to Farquhar et al [68]. The upper fully expanded healthy leaves were selected for measuring the respective parameters between 10 a.m. and 12 p.m. Three leaves per pot and 9 leaves per treatment were selected and measurements were performed on a leaf area of 1.7 cm^2^ at 420 ppm of reference CO_2_ and 1000 mol/m^2^s of photosynthetic active radiation (PAR) after 45th day of sowing [[65], [66]]. Fresh leaves were collected in the early morning to calculate the relative water contents. Leaf turgidity was obtained by immersing the leaf sample in distilled water; after an overnight immersion, the turgid weight was measured by weighing. The leaf sample was oven dried at 75 °C for 1 day to get dry weight. The following formula was used to calculate the relative water contents

RWC(%)=(FW−DW)/(TW−DW)×100

For enzyme analysis such as SOD, POD, CAT, and MDA data was taken at the peak vegetative growth stage (80 days after sowing) from four selected plants’ leaves from each replication of both normal and saline-sodic soils. To analyze the CAT activity procedure described by Faillace et al. [30] was followed; 50 mM potassium phosphate-buffer (pH 7) comprising 0.1 mM EDTA and 1% PVP was used to homogenize the leaf sample (0.6 g) and the centrifugation of the mixture was done at 3200 g for 20 min at 4 °C. The rate of decomposition of hydrogen peroxide radical (H_2_O_2_) into O_2_ and H_2_O determines the CAT activity measured using a spectrophotometer at 240 nm every 8 s for 8 times.

The SOD activity was determined by photochemical reduction of the nitro blue tetrazolium (NBT). The mixture composed of 50 μL crude protein, 30 mL of PBS (100 mM, pH 7.8), 2 mL of riboflavin (20 μM), 0.6 mL of EDTA (1 mM), 2 mL of NBT dye (750 μM), and 2 mL of methionine (130 mM). The mixture was placed for 15 min under ultraviolet light and a spectrophotometer was used for absorbance readings at 560 nm [31]. POD activity was determined according to Girma et al. [31], by adding 50 μL crude protein into a reaction mixture (I mL) containing 25 mL of PBS (1 mm mM, pH 7.8) and 14 μL of guaiacol (0.2%). The reaction mixture was heated and stirred followed by the addition of 9.5 μL of hydrogen peroxide (30%) after cooling. Absorbance was measured at 470 nm every 15 s for 4 times using a spectrophotometer. To determine MDA contents in a homogenized mixture of 500 mg leaf sample and 10 ml trichloroacetic acid (0.1%) was centrifuged for 15 min at 14000×*g*. 4.5 mL of 0.5% thiobarbituric acid was used for every single mL of enzyme extract. The cooling was done in an ice bath after heating for 30 min at 95 °C and then centrifuged for 10 min at 14000×*g*. The following formula was used to calculate the absorbance.

$$\text{MDA}\;\text{level}\;\left(\text{nmol}\right)=\Delta \;\left(A532\text{nm}-\;A600\text{nm}\right)\;156\times 10^{5}$$

Where A is the Absorption coefficient having a constant value of 156/mm cm [32].

### 2.5 Plant growth and yield attributes

Plants were harvested after the completion of vegetative and reproductive stages (120 days after sowing) and divided into three parts: cob, shoot, and root. All the plant parts were rinsed with deionized water to record growth as well as biomass parameters such as plant height, germination percentage, plants tissue length (root, shoot, cob), plant tissue fresh and dry weight (root, shoot, cob), and per pot grain yield. Plant tissue dry weight was measured after placing them at 65 °C for two days till constant weight was obtained.

### 2.6 Soil and plant chemical analysis

The maize plant samples were dried at 60 °C for 2 days until a constant weight was achieved and ground to make powder that was digested with concentrated HNO_3_ and HClO_4_. Plant macronutrients (Ca, Mg, K) were determined and Fe and Zn by an atomic absorption spectrometer.

Soil samples (1 kg per pot) were collected after the harvest of maize plants and used for analysis. Standard procedures of the US salinity staff lab, were followed to determine EC_e_, pH_s_, SAR, CO_3_^−2^, HCO_3_^−1^, Cl^−1^, K^+1^, Na^+1^, and Ca^2+^+Mg^2+^. The AB-DTPA extraction procedure was followed to measure the micronutrient (Fe, Zn) concentration [33].

### 2.7 Statistical analysis

The results of data were analyzed with analysis of variance (ANOVA) and tested for the Honest Significant Difference (HSD) at a 95% confidence interval by using Minitab 7. Figures were drawn by Microsoft Excel 2019 and the same software was used to calculate mean, standard deviation, and percentages.

## 3 Results

### 3.1 Growth responses of maize under salt stress and Fe sources

**Fig. 1:**
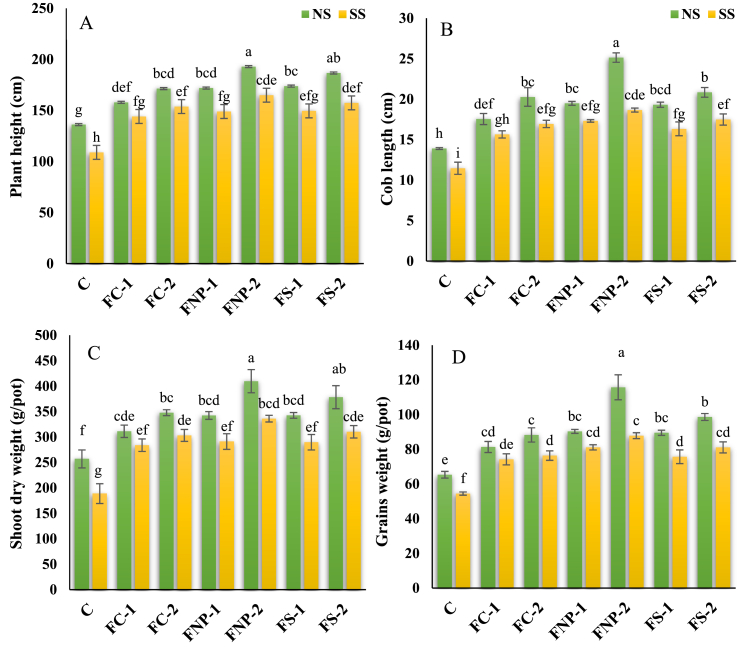
Effects of various sources of iron on maize height (A), cob length (B), shoot dry weight (C), and grain weight (D). Bars indicating the mean values, error bars showing standard error, and different lettering on bars highlighting the significance difference (p < 0.05) among the applied treatments. On X-axis C; control treatment, FC-1; iron chelate at 15 mg/kg, FC-2; iron chelate at 25 mg/kg, FNP-1; iron nanoparticles at 15 mg/kg, FNP-2; iron nanoparticles at 25 mg/kg, FS-1; iron sulphate at 15 mg/kg, FS-2; iron sulphate at 25 mg/kg, and on upper side of graphs NS; normal soil, SS; Salt-affected soil.

The residual Fe sources increased the growth parameters of maize plants at all treatment levels than control (Fig. 1A–D). It was found a significant increment in the plant height (PH), cob length (CL), root length (RL), root dry weight (RDW), shoot dry biomass (SDW), and grain biomass (GW) by 26.28, 40.10, 77.29, 24.15, 33.23, and 38.29%, respectively in normal soil, at 15 mg/kg of soil FNp concentration, compared to control. When the soil FNp concentration was increased up to 25 mgkg^−1^ in normal soil, increase was observed in the PH, CL, RL, RDW, SDW, and GW by 41.71, 80.75, 111.13, 45.68, 59.46, and 77.20%, as compared with that of the normal control. Growth parameters like PH, CL, RL, RDW, SDW, and GW also increased by other Fe sources including FC-1 (by 16.01, 26.16, 59.65, 11.79, 21.13, and 24.53%), FC-2 (by 25.89, 45.78, 73.25, 18.91, 35.36, and 24.53%), FS-1 (by 27.69, 38.85, 75.71, 21.51, 33.32, and 37.06%), and FS-2 (by 37.12, 49.87, 88.70, 34.67, 47.25, and 51.00%).

Under salt stress conditions, it was observed that residual FNp-1 caused an increase in PH, CL, RL, RDW, SDW, and GW about 36.75, 51.01, 67.42, 49.85, 54.15, and 48.93%, respectively, compared to salt-stressed control (Fig. 1A–D). While, FNp-2 caused an increment in PH, CL, RL, RDW, SDW, and GW by 51.40, 62.65, 81.26, 65.06, 78.00, and 61.24%, respectively compared with the respective control treatment. It was observed that PH, CL, RL, RDW, SDW, and GW also increased by conventional Fe sources like FC-1 (by 32.29, 36.47, 53.12, 39.44, 50.37, and 36.21%), FC-2 (by 41.24, 47.72, 63.48, 48.88, 60.55, and 40.18%), FS-1 (by 37.34, 42.40, 58.97, 42.28, 53.43, and 38.98%), and FS-2 (by 44.54, 52.49, 67.30, 55.83, 64.30, and 48.83%) compared to the respective control.

### 3.2 Chlorophyll contents and cellular responses of maize under salt stress and Fe sources

The residual Fe sources led to a significant boost in the chlorophyll concentration and photosynthetic parameters of maize crop grown under normal as well as salt stress, compared to respective controls (Table 1). In the presence of FNp-1 in normal soil an increase in chlorophyll *a*, *b*, carotenoid, total chlorophyll content, MSl, and LWRC of about 23.48, 12.44, 22.72, 23.17, 29.42, and 35.38%, respectively, was observed as compared with respective control (Table 1). Moreover, plants treated with FNp-2 caused a marked increment in chlorophyll *a*, *b*, carotenoid, total chlorophyll content, MSl, and LWRC values (by 38.36, 39.62, 58.50, 38.40, 46.04, and 52.32%) over the control. Conventional Fe sources like FC-1, FC-2, FS-1, and FS-2 also exhibited a significant increase of 11.19, 20.66, 22.38, and 33.93 % in chlorophyll *a*; 1.26, 9.88, 0.07, and 19.68% in chlorophyll *b*; 11.34, 20.35, 17.08, and 31.96% in carotenoids; 10.92, 20.36, 21.76, and 33.54% in total chlorophyll contents; 16.55, 29.48, 28.27, and 38.98% in MSI; and 23.99, 34.55, 32.09, and 46.56% in LWRC, respectively, verses to that of normal control (Table 1).

**Table 1:** Effects of various sources of iron on the photosynthetic pigments and cell strength under both normal and salt-affected soil conditions.

Treatments	Chlorophyll *a* (μg/g)		Chlorophyll *b* (μg/g)		Carotenoids (μg/g)		Total chlorophyll contents (μg/g)		RWC (%)		MSI (%)	
	NS	SAS	NS	SAS	NS	SAS	NS	SAS	NS	SAS	NS	SAS
Control	2.23 ± 0.12^e^	1.75 ± 0.03^f^	0.06 ± 0.002^cd^	0.04 ± 0.005^e^	0.59 ± 0.08^g^	0.43 ± 0.02^h^	2.29 ± 0.12^e^	1.79 ± 0.03^f^	69.04 ± 2.70^f^	58.09 ± 2.90^g^	66.78 ± 2.17^e^	52.85 ± 2.09^f^
FNp-1	2.75 ± 0.03^b^	2.47 ± 0.05^cd^	0.07 ± 0.001^bc^	0.06 ± 0.001^cd^	0.73 ± 0.02^bc^^d^	0.64 ± 0.02^c-g^	2.82 ± 0.04^b^	2.53 ± 0.05^cd^	93.47 ± 1.73^bc^	85.39 ± 1.59^cde^	86.42 ± 1.08^b^	77.63 ± 1.44^c^
FNp-2	3.08 ± 0.07^a^	2.63 ± 0.10^bc^	0.09 ± 0.004^a^	0.07 ± 0.001^bc^	0.94 ± 0.04^a^	0.70 ± 0.01^b-e^	3.17 ± 0.07^a^	2.70 ± 0.10^bc^	105.17 ± 0.97^a^	93.10 ± 1.05^bc^	97.52 ± 1.35^a^	84.05 ± 1.64^b^
FC-1	2.48 ± 0.10^cd^	2.26 ± 0.10^de^	0.06 ± 0.003^cd^	0.06 ± 0.001^c^^d^	0.66 ± 0.01^c-g^	0.60 ± 0.01^fg^	2.54 ± 0.10^cd^	2.32 ± 0.10^de^	85.61 ± 3.35^cde^	78.10 ± 3.37^e^	77.83 ± 3.05^c^	69.46 ± 2.39^de^
FC-2	2.69 ± 0.12^bc^	2.39 ± 0.06^de^	0.07 ± 0.003^bc^	0.06 ± 0.002^cd^	0.71 ± 0.04^bcd^	0.63 ± 0.01^d-g^	2.76 ± 0.13^bc^	2.45 ± 0.06^de^	92.90 ± 4.32^bc^	83.39 ± 3.20^de^	86.46 ± 1.33^b^	75.81 ± 2.91^c^
FS-1	2.73 ± 0.04^b^	2.37 ± 0.04^de^	0.06 ± 0.006^cd^	0.06 ± 0.003^cd^	0.69 ± 0.01^b-f^	0.61 ± 0.02^efg^	2.79 ± 0.04^b^	2.43 ± 0.04^de^	91.20 ± 1.71^cd^	79.69 ± 4.16^e^	85.66 ± 1.41^b^	73.55 ± 2.07^cd^
FS-2	2.98 ± 0.03^a^	2.47 ± 0.10^cd^	0.08 ± 0.002^b^	0.07 ± 0.002^bc^	0.78 ± 0.01^bc^	0.63 ± 0.03^d-g^	3.06 ± 0.03^a^	2.53 ± 0.10^cd^	101.19 ± 2.56^ab^	85.66 ± 2.90^cde^	92.81 ± 1.00^a^	77.57 ± 3.05^c^

In the table FNP-1; iron nanoparticles at 15 mg/kg, FNP-2; iron nanoparticles at 25 mg/kg, FC-1; iron chelate at 15 mg/kg, FC-2; iron chelate at 25 mg/kg, FS-1; iron sulphate at 15 mg/kg, FS-2; iron sulphate at 25 mg/kg. NS; normal soil, SS; Salt-affected soil. Values are showing mean ± standard deviation and different lettering highlighting the significant difference among the applied treatments.

In contrast, under salt stress conditions residual FNp and conventional sources alleviated the adverse effects of salinity stress and showed more obvious results in comparison with the normal soil. The FNp-1 showed an increase in chlorophyll *a* content by 40.92%, chlorophyll *b* content by 54.41%, carotenoid by 49.07%, total chlorophyll content by 41.23%, MSl by 46.88%, LWRC by 47.00%, respectively, observed in FNp-1 under salt-affected soil (Table 1). The harmful effect of salt-stress on chlorophyll contents and photosynthetic parameter was efficiently mitigated by FNp-2 and maize plants exhibited higher levels of chlorophyll *a* content by 49.91%, chlorophyll *b* content by 67.18%, carotenoid by 62.48%, total chlorophyll content by 50.31%, MSl by 59.02%, LWRC by 60.27% than the plants grown under salt-affected soil without treatment application. On the other hand, conventional Fe sources like FC-1, FC-2, FS-1, and FS-2 showed an increase of 28.89, 36.28, 35.35, and 40.82% in chlorophyll *a*; 43.57, 51.47, 44.10, and 59.67% in chlorophyll *b*; 39.79, 46.01, 41.33, and 46.73% in carotenoids; 29.22, 36.63, 35.55, and 41.26% in total chlorophyll contents; 31.42, 43.43, 39.16, and 46.77% in MSI; and 34.44, 43.54, 37.18, and 47.45% in LWRC, respectively, verses to that of respective control (Table 1).

### 3.3 Physiological and gas exchange responses of maize under salt stress and Fe sources

**Fig. 2:**
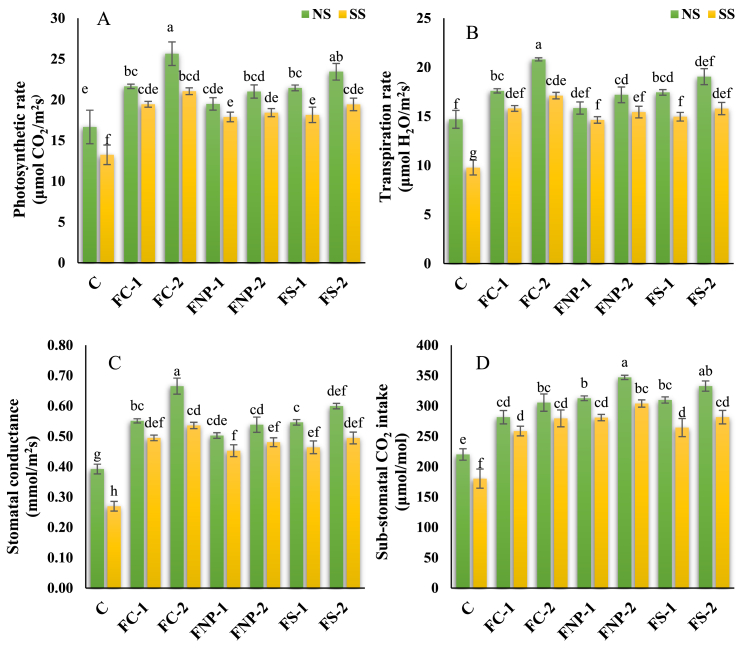
Effects of various sources of iron on the photosynthetic rate, umol m^-2^ s^-1^ (A), transpiration rate, mmol m^-2^ s^-1^ (B), stomatal conductance, mol m^-2^ s^-1^ (C), and sub-stomatal CO_2_ intake (D). Bars indicating the mean values, error bars showing standard error, and different lettering on bars highlighting the significance difference (p < 0.05) among the applied treatments. On X-axis C; control treatment, FC-1; iron chelate at 15 mg/kg, FC-2; iron chelate at 25 mg/kg, FNP-1; iron nanoparticles at 15 mg/kg, FNP-2; iron nanoparticles at 25 mg/kg, FS-1; iron sulphate at 15 mg/kg, FS-2; iron sulphate at 25 mg/kg, and on upper side of graphs NS; normal soil, SS; Salt-affected soil.

The application of FNp positively affected the physiological and gaseous attributes of maize plants regardless of whether the plants were grown under salt-affected and normal soil (Fig. 2A–D). Without salt stress, the application of FNp-1 increased the photosynthetic rate by 29.9%, transpiration rate by 19.73, stomatal conductance by 40.5%, and sub-stomatal CO_2_ concentration by 42.0%. This same treatment under salinity, increased the photosynthetic rate by 46.8%, transpiration rate by 61.7%, stomatal conductance by 83.53%, and sub-stomatal CO_2_ concentration by 68.6% compared with the respective control. The best response was shown by the FNp-2 regardless of whether the plants were grown under normal or salt-affected soil. The photosynthetic rate, transpiration rate, stomatal-conductance, and sub-stomatal CO_2_ levels were increased by 54.0, 41.6, 69.6, and 57.7% respectively, with the application of FNp-2 when there was no salt stress; however, under salt stress, the usage of the same treatment delivered the highest contents of these parameters showed 58.9, 75.0, 98.7, and 68.6% increase respectively, compared to control.

The conventional Fe sources also significantly increased the gas exchange attributes regardless of the stress condition (Fig. 2A–D). Without salt stress, the application of FC-1 and FC-2 increased the photosynthetic rate by 17.0% and 26.1, transpiration rate by 7.8% and 17.0%, stomatal conductance by 28.1%, and 37.27%, sub-stomatal CO_2_ concentration by 27.9%, and 38.8%, respectively. While, under salinity, the same treatments increased the photosynthetic rate by 35.03% and 39.03%, transpiration rate by 49.67% and 57.88%, stomatal conductance by 67.9%, and 78.2%, sub-stomatal CO_2_ concentration by 43.5%, and 55.0%, respectively. Without salt stress, the application of FS-1 and FS-2 increased the photosynthetic rate by 28.8% and 40.7%, transpiration rate by 18.7% and 29.6%, stomatal conductance by 39.2%, and 52.9%, sub-stomatal CO_2_ concentration by 40.8%, and 51.2%, respectively, while, under salinity, the same treatments increased the photosynthetic rate by 37.0% and 46.7%, transpiration rate by 53.0% and 61.6%, stomatal conductance by 72.0%, and 83.4%, sub-stomatal CO_2_ concentration by 46.6%, and 56.2%, respectively.

### 3.4 Antioxidant enzyme activities under salt stress and Fe sources

**Fig. 3:**
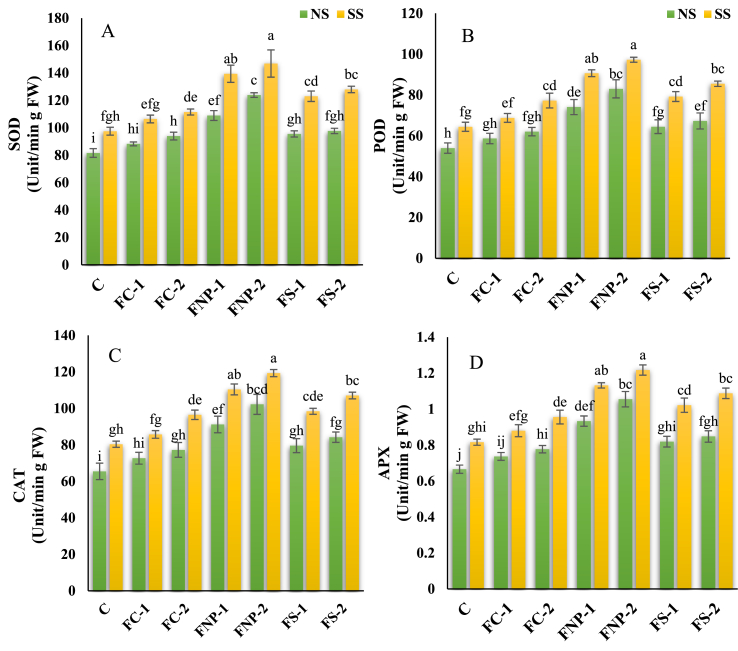
Effects of various sources of iron on the superoxide dismutase (SOD) (A), peroxide dismutase (POD) (B), catalase (CAT) (C), and ascorbate peroxidase (APX) (D). Bars indicating the mean values, error bars showing standard error, and different lettering on bars highlighting the significance difference (p < 0.05) among the applied treatments. On X-axis C; control treatment, FC-1; iron chelate at 15 mg/kg, FC-2; iron chelate at 25 mg/kg, FNP-1; iron nanoparticles at 15 mg/kg, FNP-2; iron nanoparticles at 25 mg/kg, FS-1; iron sulphate at 15 mg/kg, FS-2; iron sulphate at 25 mg/kg, and on upper side of graphs NS; normal soil, SS; Salt-affected soil.

Salt stress considerably affected the enzymatic activity by inducing secondary oxidative stress in maize plants. The application of Fe sources has significant effects on the antioxidant enzymes of the leaf (Fig. 3A–D). However, the maximum improvement in the activity of SOD (51.78, 50.61%), POD (53.78, 51.01%), CAT (56.08, 48.54%), and APX (58.44, 49.18%) was observed in the plants treated with FNp-2 grown under normal as well as salt stressed soil respectively when compared with the respective controls. While, minimum improvement in the activity SOD (8.17, 9.14%), POD (8.7, 6.75%), CAT (11.01, 6.67%), and APX (10.67, 7.85%) was observed in the plants treated with FC-1 under the same conditions. Generally, the treatments affected the antioxidant enzyme activity in the following trend FNp-2 > FNp-1 > FS-2 > FS-1 > FC-2 > FC-1 under both normal as well as salt-stressed conditions.

### 3.5 Ionic composition of soils and maize plants under salt stress and Fe sources

**Fig. 4:**
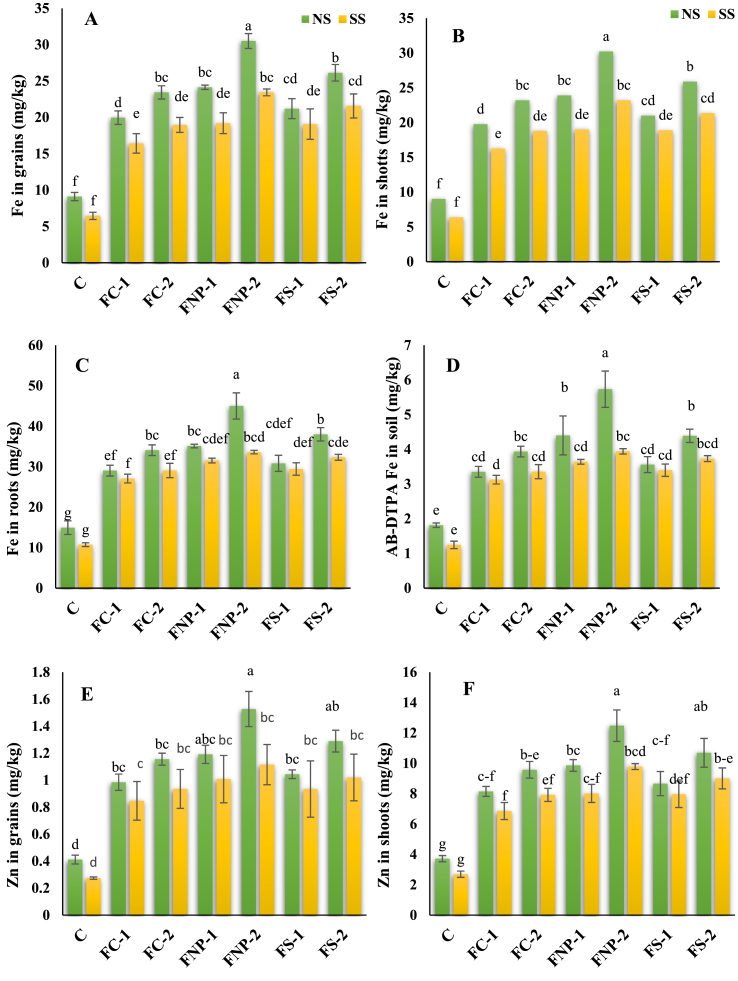

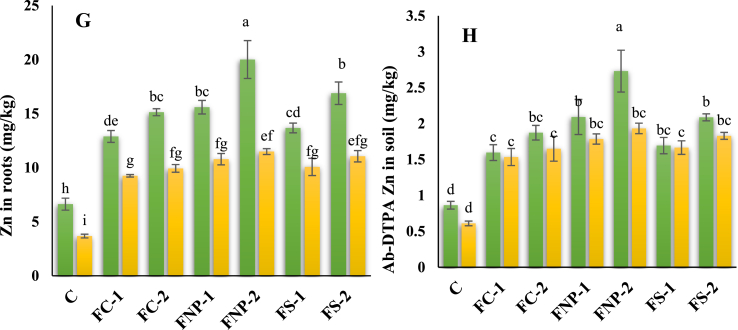
Effects of various sources of iron on the concentration of iron in grains (A), shoots (B), roots (C), soil (AB-DTPA) (D); zinc in grains (E), shoots (F), roots (G) and soil (AB-DTPA) (H). Bars indicating the mean values, error bars showing standard error, and different lettering on bars highlighting the significance difference (p < 0.05) among the applied treatments. On X-axis C; control treatment, FC-1; iron chelate at 15 mg/kg, FC-2; iron chelate at 25 mg/kg, FNP-1; iron nanoparticles at 15 mg/kg, FNP-2; iron nanoparticles at 25 mg/kg, FS-1; iron sulphate at 15 mg/kg, FS-2; iron sulphate at 25 mg/kg, and on upper side of graphs NS; normal soil, SS; Salt-affected soil.

Interacting effects of salt stress and Fe sources were found to be statistically significant on Fe and Zn concentration in roots, shoots, grains, and soils. The maximum Fe concentration in soil (216.20%) was observed in FNp-2 in the absence of salt stress compared to the respective control followed by FNp-1(142.72%) > FS-2(142.07%) > FC-2(117.08%) > FS-1(96.33%) > FC-1(84.91%). Under salt stress conditions, an increasing trend in the following sequence; FNp-2(216.33%) > FS-2(199.52%) > FNp-1(192.18%) > FS-1(172.66%) > FC-2(169.41%) > FC-1(150.84%) was observed for Fe content of soil compared to the respective control (Fig. 4A–H).

The maximum Fe concentration (201.14%) in maize roots was observed in FNp-2 followed by FS-2 (154.22%) > FNp-1 (134.83%) > FC-2 (127.98) > FS-1 (106.19%) > FC-1 (94.20%) (Fig. 4C). In normal soil; however, under salt stress conditions the Fe concentration in roots showed the following trend FNp-2 (213.00%) > FS-2 (201.02%) > FNp-1 (193.64%) > FS-1 (174.03%) > FC-2170.76%) > FC-1 (152.09%) compared to the respective control. The shoots and grains of maize plants showed the same trend FNp-2 (234.83, 234.83%) > FS-2 (186.86, 186.86%) > FNp-1 (164.97, 164.97%) > FC-2 (157.24, 157.24%) > FS-1 (132.66, 132.66%) > FC-1(119.13, 119.13%) respectively, under normal soil, however, under salt stress conditions the trend was changed to FNp-2 (263.27, 263.27%) > FS-2 (234.29, 234.29%) > FNp-1 (197.57, 197.57%) > FS-1 (195.57, 195.57%) > FC-2 (193.95, 193.95%) > FC-1(154.52, 154.52%) respectively, than respective control.

**Fig. 5:**
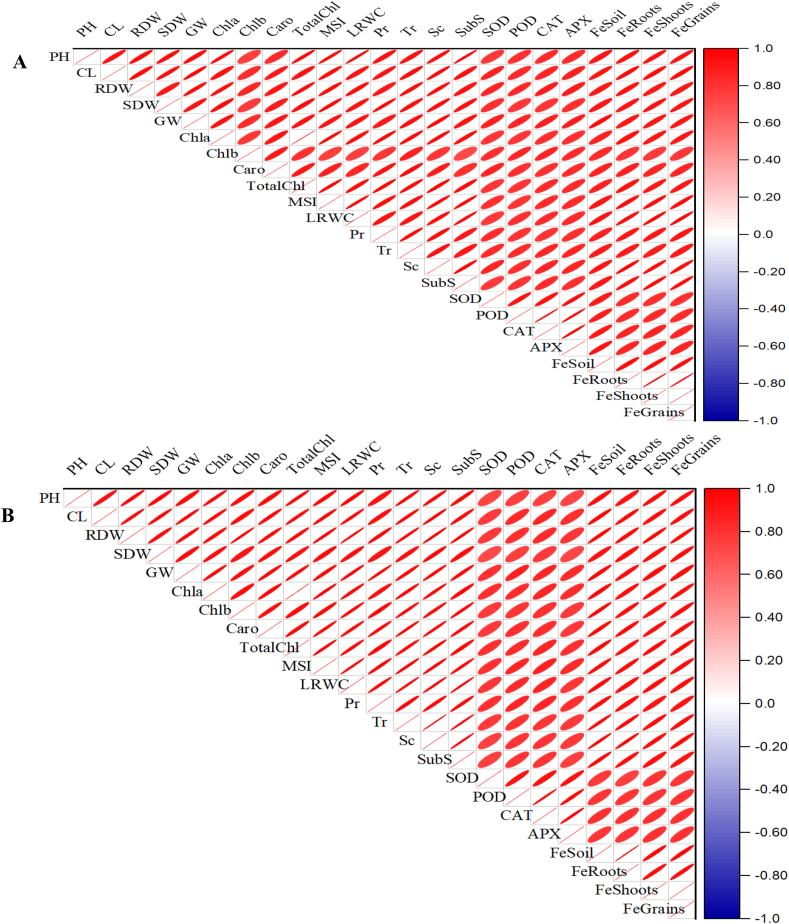
Pearson correlation analysis depicting strength of relation among various maize parameters under investigation. The parameters include plant height (PH), cob length (CL), root dry weight (RDW), shoot dry weight (SDW), grain weight (GW), chlorophyll *a* (Chla), chlorophyll *b* (Chl) carotenoids (Caro), total chlorophyll contents (TotalChl), membrane stability index (MSI), leaf relative water contents (LWRC), photosynthetic rate (Pr), transpiration rate (Tr), stomatal conductance (Sc), sub-stomatal CO_2_ intake (SubS), superoxide dismutase (SOD), peroxide dismutase (POD), catalase (CAT), ascorbate peroxidase (APX), iron in soil (FeSoil), iron in roots (FeRoots), iron in shoots(FeShoots), and iron in grains (FeGrains). A; correlation analysis in normal soil and B; correlation analysis in salt-affected soil.

The highest Zn concentration was recorded for the plants treated by FNp-2 under normal as well as salt-stressed conditions followed by FS-2 > FNp-1 > FS-1 > FC-2 > FC-1 with few exceptions. Under normal and salt stress conditions, maximum Zn concentration (270.92 and 306.88%, respectively) in grain was found in the FNp-2 treated plants followed by FS-2 (213.14, 272.23%) > FNp-1 (189.36, 267.78%) > FC-2 (180.62, 241.25%) > FS-1 (153.54, 240.93%) > FC-1(139.17, 209.01%). Under normal conditions, shoot and root recorded the maximum Zn concentration (235.61, 201.96%) in FNp-2 treated plants followed by FS-2 (187.55, 154.87%) > FNp-1 (165.18, 128.34%) > FC-2 (157.47, 128.34%) > FS-1 (133.05, 106.38%) > FC-1(119.16, 94.55%). However, under saline conditions shoot and root followed slightly different trends FNp-2 (263.27, 213.00%) > FS-2 (234.29, 201.15%) > FNp-1 (197.57, 193.80%) > FS-1 (195.57, 174.31%) > FC-2 (193.95, 170.50%) > FC-1(154.52, 151.94%). The Zn concentration in the normal soil were found in the following trend FNp-2 (216.26%) > FNp-1 (142.32%) > FS-2 (141.75%) > FC-2 (117.00%) > FC-1 (84.92%) > FS-1(96.19%) while in the saline soil, the trend was different FNp-2 (216.90%) > FS-2 (199.97%) > FNp-1(192.72%) > FS-1 (173.10%) > FC-2 (170.47%) > FC-1(151.58%). The correlation analysis of studied parameters in plants (Fig. 5A–B) showed the positive and negative relationships among the studied parameters. The correlation analysis also indicated the strength of relationship among the studied parameters in plants.

### 3.6 Chemical properties of soils under salt stress and Fe sources

Interacting effects of salinity and Fe sources were found to be statistically significant on soil SAR, EC_e_, Na, and Ca + Mg as shown in Table 2. The Fe sources decreased SAR, EC_e_, and Na under normal and saline conditions, while, Ca + Mg concentration increased under the same conditions, as compared with the control. The maximum reduction in soil SAR, EC_e_, and Na was at FNP-2, and the minimum reduction in these traits (SAR, EC_e_, and Na was observed in FC-1 under both normal and saline conditions respectively, as compared with the control. The maximum Ca + Mg were observed in FNP-2, and the minimum observed in control under both normal and saline conditions.

**Table 2:** Effects of various sources of iron on the chemical properties of in pots under both normal and salt-affected soil conditions.

Treatments	SAR {(mmol/L)^1/2^}		EC (dS/m)		Na (me/L)		Ca + Mg (me/L)	
	NS	SAS	NS	SAS	NS	SAS	NS	SAS
Control	14.53 ± 0.18^c^	28.20 ± 1.04^a^	3.94 ± 0.03^de^	7.89 ± 1.02^a^	27.80 ± 0.30^e^	61.00 ± 3.13^a^	7.32 ± 0.31^g^	9.36 ± 0.41^ef^
FNp-1	9.62 ± 0.07^e^	9.49 ± 0.68^e^	3.54 ± 0.02^ef^	5.33 ± 0.24^bc^	21.11 ± 0.07^g^	24.88 ± 1.68^efg^	9.63 ± 0.16^ef^	13.75 ± 0.49^b^
FNp-2	7.33 ± 0.55^f^	12.34 ± 0.14^d^	3.44 ± 0.06^ef^	5.09 ± 0.24^bc^	16.45 ± 1.30^h^	33.91 ± 1.45^d^	10.06 ± 0.07^e^	15.21 ± 1.47^a^
FC-1	11.33 ± 0.28^d^	15.21 ± 0.16^bc^	3.74 ± 0.07^de^	5.56 ± 0.25^b^	23.29 ± 0.56^fg^	36.54 ± 0.90^d^	8.45 ± 0.02^fg^	11.54 ± 0.35^d^
FC-2	11.44 ± 0.15^d^	15.32 ± 0.49^bc^	3.58 ± 0.04^ef^	4.90 ± 0.09^bc^	24.23 ± 0.15^efg^	37.80 ± 1.35^cd^	8.97 ± 0.13^ef^	12.17 ± 0.22^cd^
FS-1	11.46 ± 0.15^d^	16.29 ± 0.49^b^	3.20 ± 0.01^ef^	4.61 ± 0.16^cd^	24.57 ± 0.33^efg^	40.68 ± 0.70^bc^	9.20 ± 0.04^ef^	12.48 ± 0.33^bcd^
FS-2	12.08 ± 0.25^d^	16.79 ± 0.79^b^	2.69 ± 0.14^f^	3.61 ± 0.18^e^	26.02 ± 0.66^ef^	43.10 ± 2.04^b^	9.28 ± 0.08^ef^	13.17 ± 0.17^bc^

In the table FNP-1; iron nanoparticles at 15 mg/kg, FNP-2; iron nanoparticles at 25 mg/kg, FC-1; iron chelate at 15 mg/kg, FC-2; iron chelate at 25 mg/kg, FS-1; iron sulphate at 15 mg/kg, FS-2; iron sulphate at 25 mg/kg. NS; normal soil, SS; Salt-affected soil. Values are showing mean ± standard deviation and different lettering highlighting the significant difference among the applied treatments.

## 4 Discussion

### 4.1 The influence of salt stress and Fe sources on maize growth

Among the most common effects of salinity on plants, growth reduction is a major constraint in plant production that results in yield losses [[1], [2], [64], [67]]. Our results revealed that salinity had deleterious effects on maize growth parameters such as PH, CL, RT, RDW, SDW, and GW compared to those which were not subjected to salinity (Fig. 1A–D). However, the residual FNp particularly at the rate of 25 mg/kg, alleviated the deleterious effects of salinity in the aforementioned plant parameters (Fig. 1A–D). A higher concentration of salts in the soil solution decreases the availability of water to the plant roots due to lower osmotic potential in the seedbed resulting in limited plant growth, reduced seed germination, and poor seedling establishment [34,[67]]. Under saline conditions, Na influx is increased compared to K ions due to the similarity in the hydrated Na and K ions and it becomes difficult for influx pathways to differentiate between Na and K ions resulting in Na ions toxicity [3]. Furthermore, ionic toxicity due to excessive accumulation of Na ions in the cell cytoplasm results in the disrupted cell membrane, reduced energy production, and restricted anabolic processes, ultimately affecting cell division, elongation, biomass accumulation, and hence, plant growth characteristics. Recently, Javed et al. [8] have reported that extent of reduction in plant growth attributes under salt stress depends on the source of salt and its concentration. The results of our experiment were consistent with the findings of Kaur et al. [35] and Isik [27], showing that the overall germination percentage and early seedling growth were found to be affected by salinity. In the current study, Fe sources decreased the Na accumulation and enhanced tolerance of salt stress in maize plants (Table 2). In general, Fe is an essential micronutrient which perform many roles in a large number of biochemical and physiological ways in plants. The competition between Na and Fe uptake results in the limited influx of Na by maize roots. Wong et al. [36] reported that Fe decreased the Na in wheat plants under salt stress conditions. High salt concentrations in the root zone enhance the Na uptake and translocation, however, the application of Fe_2_O_3_ nanoparticles stimulated root pumps activity in ajowan plants and thus reduced the entrance of Na ions compared to the Fe leading to a better plant growth [19]. Zia-ur-Rehman et al. [28] reported an increase in root activity, seed germination, seedling growth, and watermelon biomass as well as resistance to the environmental stresses upon exposure to the different concentrations of nano-Fe.

### 4.2 Photosynthetic and gaseous exchange responses of maize plants

Photosynthetic pigments and proteins play a key role in performing foliar gas exchange, whole-plant growth, and crop performance by harvesting light energy into chemical energy [37[[65], [68], [69]]]. Salt-affected soils are deficient in micronutrients as high pH decreases their availability after reaction with ions including Na, Cl, and SO_4_^2−^ [[5], [67]]. In high-pH soils, the availability of Fe is limited due to the formation of insoluble hydroxides and oxide complexes of Fe on the soil surface [38]. The reduced availability of Fe inhibited photosynthesis as Fe is an important part of several enzymes that are involved in the synthesis of chlorophyll [[39], [69], [70]]. Moreover, Fe deficiency results in chlorosis and vein yellowing in young leaves [[40], [71]]. Damage to the photosynthetic machinery from higher Na^+^ accumulation and lower levels of K and Mg ions in the plant leaves [18]. Deficient biosynthesis of photosynthetic pigments and proteins under salt stress leads to reduced plant growth and development. Our results revealed that salt stress significantly reduced the photosynthesis, RWC, and MSI of maize plants when compared to the respective control. Nevertheless, residual FNp displayed a substantial capability to improve the photosynthetic process by regulating the production of chlorophyll *a*, chlorophyll *b*, carotenoids, MSI, and RWC as well as alleviating the negative impacts of salt stress in maize seedlings. It was also observed that the response of FNp was more obvious in stressed plants compared to non-stressed plants. The suppression of ROS production, sodium ion toxicity, and maintenance of chloroplast functionality under residual FNp highlighted some amelioration for photosynthetic pigments, better cellular water availability and plausible Na^+^ ions extrusion [18,23,38,41]. Furthermore, residual FNp reduced leaf abscission and enhanced photosynthetic activity, thus improving plant physiological performance. Similarly, in tomato cultivars, an increase in the concentration of chlorophyll *a*, chlorophyll *b*, and carotenoids were reported following the application of FNp under salinity stress [42]. The FNp application to *Eucalyptus tereticornis* grown under high salinity has shown huge potential to improve plant physiological performance by acting as a nano supplement [7]. Therby-Vale et al. [40] reported that the use of FNp in the micropropagation of moringa under salt stress that improved plant vigor due to enhanced chlorophyll *a*, chlorophyll *b*, and carotenoid contents. A favourable plant water status has been observed under residual FNp that can be attributed to improved plant growth, chlorophyll contents, and photosynthetic process. In the current study, under salt stress, decrease in the aforementioned parameters has been depicted in our results. However, residual FNp and conventional sources at both concentrations i.e., 15 mg/kg and 25 mg/kg alleviated the salinity stress and showed a significant improvement in all physiological traits (Fig. 2A–D). Similarly, Manaa et al. [43] and Gohari et al. [37] demonstrated that prolonged exposure to salt stress causes some morphological changes in chloroplasts such as swollen thylakoids, accumulation of starch granules and plastoglobuli and disrupted envelope in *Chenopodium quinoa* and *Thellungiella salsuginea*. Salt stress-induced morphological changes in stomata and mesophyll cells would alter intracellular CO_2_ and leaf-level CO_2_ flux [44]. In another study, results depicted that salt stress did not disturb the intracellular CO_2_ but decreased stomatal conductance, transpiration rate, and photosynthetic CO_2_ gain in halfa grass [45]. In addition to reduced photosynthetic activity, high Na^+^ accumulation increased the non-photochemical quenching attributes and therefore decreasing the efficiency of photosystem-II and photochemical quenching parameters [46]. Furthermore, lower photosynthetic activities in maize under salt stress was reported recently and attributed to poor osmoregulation, insufficient K intake, and concomitant stomatal closure [47].

### 4.3 Antioxidant enzymes activity in maize plants

The application of Fe nanoparticles mitigates the damage of abiotic stress (heavy metals and salt stress) by lowering the production of oxidative stress indicators and increasing antioxidant enzymes activity that stabilizes the photosynthetic machinery and improved the net photosynthetic rate [15,48]. Plants subjected to salt stress led to the excessive production of ROS that greatly depends upon the alteration in growth conditions, the plant's ability to adapt to changes in energy imbalance and the severity and duration of stress [49]. Excessive ROS production results in oxidative stress various cell compartments by oxidizing lipids, proteins, RNA, and DNA, leading to disrupted cell functions and membrane integrity [45]. However, ROS production is balanced by the activation of the antioxidant defense system including several enzymes [12]. In the current study, oxidative damage induced by salt stress in maize plants can be ameliorated through enhanced antioxidant enzyme activity (Fig. 3A–D). One significant analysis that results from this work is that the residual FNp-2 enhanced the antioxidant enzyme activity in both the presence and absence of salt stress. Previous studies have revealed that FNp decreased ROS production and improved the antioxidant enzyme (CAT, POD, SOD, and APX) activity in plants by improving the transpiration rate and chlorophyll contents under abiotic stress conditions which was the result of upregulation of signaling genes [24,[50], [51], [52]]. Moreover, FNp may reduce oxidative damage to plants by solving the problem of Fe deficiency and reducing proline and MDA contents [53]. Application of nano-Fe resulted in altered plant metabolic activities such as increased antioxidant enzymes activity and phytohormone contents in plants [21,41]. Yasmeen et al. [54] reported increased SOD activity in the seeds treated with FNp that contribute to oxidative stress scavenging under unfavorable conditions.

### 4.4 Nutrient uptake by maize plant under salts stress and various sources of Fe

Salinity affects plant growth and physiological activity by imposing osmotic stress and hampers nutrient uptake [[2], [64], [67]]. In this work, ionic uptake and built-up in the leaves and roots of maize plants have gained special focus. Interesting, our results revealed a significant effect of salt stress on ions such as Zn, and Fe (Fig. 4A–H). Specifically, the highly significant results were observed for Zn, and Fe in response to residual FNp-2 compared to the control. In general, salinity alters ion uptake and interactions among different micronutrients; ultimately causing deficiency issues (e.g., Fe and Zn), athereby affecting plant physiology [[55], [71],]. Zeiner et al. [56] noted that Fe uptake and translocation were drastically decreased in Chinese cabbage when subjected to salt stress. Similarly increased accumulation of total shoot Fe and decreased total shoot Zn has been observed by Zahra et al. [57] in wheat plants under salt stress and different levels of phosphorous supply. The application of Fe lowered the uptake of Zn as they appeared to have an antagonistic effect in absorption by plants [42,58]. Inhibited upregulation of Fe and Zn transporters might be the major reason behind Fe and Zn deficiency symptoms in salt-stressed plants [[59], [60], [63]]. In the current study, the external use of Fe plausibly repressed the accumulation of Na by maize tissues and improving the tolerance against salt stress. Our results were consistent with a study where exogenous application of Fe alleviated Na and other toxic ions-linked injury in *Moringa peregrina* plants under salt stress [61]. Similarly, the exogenous application of FNp reduced the uptake of sodium by *Aloe vera*under salinity stress [62]. Specifically, the whole-plant nutritional balance appeared to be maintained by the rapid absorption of Fe due to the small size of applied FNp [25]. At the mechanistic level, FNp could enhanced the activity of H^+^ pumps (H^+^-ATPase and H^+^-PPase) that regulate the absorption of nutrients in saline condtion and preventing the active uptake of Na as well as its translocation [19].

## 5 Conclusion

The present study concluded that FNp application at a higher residual dosage (25 mg/kg) significantly enhanced the maize growth and yield grown on saline-sodic soil. Higher rate of FNp alleviated salt stress by enhancing the physiological and biochemical attributes of maize including photosynthetic components, membrane stability, antioxidant enzymes activities and uptake of essential nutrients. Conventional sources of Fe such as Fe-EDTA and FeSO_4_ also increased the yield of maize in both normal and saline soils. FNp increased the tolerance of maize against salinity by increasing the antioxidants activities of SOD, POD, CAT, while decreasing MDA contents. Interestingly, the application of FNp synergized the uptake of K, Zn, Fe, Ca and Mg by maize under salt stress. These favourable findings would support further investigation on the applications of NPs at different growth stages to alleviate different climatic stresses in plants.

## Funding

This work was funded by the Institutional Fund Projects under grant no. (IFPIP: 737-130-1443), Ministry of Education in Saudi Arabia.

## Data availability statement

Most of the data are available in all Tables and Figures of the manuscripts. Moreover, the data will available on request.

## CRediT authorship contribution statement

**Hameed Alsamadany:** Writing – original draft, Resources, Funding acquisition, Formal analysis. **Sidra Anayatullah:** Writing – review & editing, Software, Formal analysis, Data curation. **Muhammad Zia-ur-Rehman:** Writing – review & editing, Resources, Project administration, Investigation, Funding acquisition, Conceptualization. **Muhammad Usman:** Writing – review & editing, Software, Methodology, Data curation. **Talha Ameen:** Writing – review & editing, Visualization, Formal analysis, Data curation. **Hesham F. Alharby:** Writing – review & editing, Visualization, Software. **Basmah M. Alharbi:** Writing – review & editing, Validation, Software, Investigation. **Awatif M. Abdulmajeed:** Writing – review & editing, Software, Investigation. **Jean Wan Hong Yong:** Writing – review & editing, Validation, Resources, Project administration, Funding acquisition, Conceptualization. **Muhammad Rizwan:** Writing – review & editing, Writing – original draft, Conceptualization.

## Declaration of competing interest

The authors declare that they have no known competing financial interests or personal relationships that could have appeared to influence the work reported in this paper.
