# Supplementary material for: Residual efficiency of iron-nanoparticles and different iron sources on growth, and antioxidants in maize plants under salts stress: life cycle study
Source: Heliyon. 2024 Apr 1;10(7):e28973. doi: 10.1016/j.heliyon.2024.e28973 (PMC11004812; doi:10.1016/j.heliyon.2024.e28973)
Supplement: Multimedia component 1 [file mmc1.docx]

**Supplementary Figure 1**. Pictorial view of experiment.
